# The correlation between traditional Chinese medicine constitution and prediabetes: a systematic review and meta-analysis

**DOI:** 10.3389/fendo.2026.1678799

**Published:** 2026-02-03

**Authors:** Liqian Chen, Yun Wang, Xinhui Xie, Zhiyi Zhou, Lu Xie, Jiaxin Cai, Hao Li, Xinyu Zhang, Yijin Ke, Qingzi Li, Zhiqi Wu, Xiaoshan Zhao, Wenying Wang

**Affiliations:** 1 The Affiliated Guangzhou Hospital of Traditional Chinese Medicine of Guangzhou University of Chinese Medicine, Guangzhou, Guangdong, China; 2 The Affiliated Traditional Chinese Medicine Hospital of Guangzhou Medical University, Guangzhou, Guangdong, China; 3 The Clinical School of Integrated Traditional Chinese and Western Medicine, Guangzhou Medical University, Guangzhou, Guangdong, China; 4 School of Traditional Chinese Medicine, Southern Medical University, Guangzhou, Guangdong, China

**Keywords:** balance constitution, constitution, meta-analysis, phlegm-damp constitution, prediabetes, traditional Chinese medicine

## Abstract

**Objectives:**

The aims of this study were to investigate the distribution of traditional Chinese medicine (TCM) constitution types in individuals with prediabetes and to identify high-risk constitutions, thereby providing an evidence-based foundation for the prevention and treatment of prediabetes.

**Methods:**

We systematically searched PubMed, Embase, Web of Science, the Cochrane Library, and four Chinese databases for literature examining the association between prediabetes and TCM constitution types. A single-proportion meta-analysis of cross-sectional studies and a comparative meta-analysis of case–control studies comparing individuals with prediabetes and the general population were performed using the Stata17.0 software. Effect sizes were expressed as odds ratios (ORs) with 95% confidence intervals (CIs). Study quality was assessed independently by two reviewers. The primary outcomes included the distribution of TCM constitution types in the prediabetes population and the comparative ORs between groups.

**Results:**

A total of 30 cross-sectional studies and 5 case–control studies, involving 8,469 participants, were included. Among individuals with prediabetes, the pooled prevalence rates of phlegm-dampness constitution (PDC), balanced constitution (BC), yin-deficiency constitution (YIDC), qi-deficiency constitution (QDC), and damp-heat constitution (DHC) were 20% (95% CI: 16%–24%), BC 16% (10%–22%), 12% (10%–15%), 11% (9%–14%), and 10% (7%–13%), respectively. Meta-analysis of case–control studies indicated that the ORs for prediabetes risk in individuals with PDC, qi-stagnation constitution (QSC), QDC, and YIDC were PDC 2.49 (95CI%: 1.27-4.87), 2.03 (1.06–3.90), 1.78 (1.11–2.84), and 1.52 (1.09–2.10), respectively, while the OR for BC was 0.45 (0.30–0.66). Subgroup analyses revealed variations in TCM constitution distribution across regions and age groups, as well as difference associated with study quality.

**Conclusion:**

PDC, YIDC, QDC, DHC, and BC are the most common TCM constitution types (prevalence ≥10%) observed in individuals with prediabetes. PDC, QDC, YIDC, and QSC may represent risk factors for prediabetes, whereas BC appears to be a protective factor. Further high-quality case–control and cohort studies are warranted to strengthen the evidence regarding the relationship between prediabetes and TCM constitution types.

**Systematic Review Registration:**

https://www.crd.york.ac.uk/prospero/, identifier CRD42024607164.

## 1 Introduction

Prediabetes is an intermediate stage between normal glucose regulation and overt diabetes. According to the World Health Organization (WHO), prediabetes can be classified as three states, impaired fasting glucose (IFG), impaired glucose tolerance (IGT), and a combination of both. IFG is defined as a fasting plasma glucose (FPG) level of 6.1–6.9 mmol/L and IGT is defined as a blood glucose level of 7.8–11.0 mmol/L 2 h after oral glucose tolerance test (OGTT) (1). Studies indicate that individuals with prediabetes exhibit increased arterial stiffness—a powerful independent predictor of cardiovascular events and all-cause mortality (2–4). Prediabetes is also found to be associated with increased risk of diabetes, cardiovascular events, chronic kidney disease, cancer and cognitive decline (5–8). Despite these significant risks, prediabetes often remains undiagnosed, preventing timely intervention. Coupled with modern lifestyles, this has led to a rising global prevalence. In 2021, the global prevalence of IGT was 9.1% (464 million) and is projected to rise to 10.0% (638 million) by 2045. Similarly, the prevalence of IFG was 5.8% (298 million) in 2021 and is expected to reach 6.5% (414 million) by 2045, with the largest relative increase projected in low-income countries (9). Therefore, it is crucial to enhance the screening of high-risk populations, improve the identification and management of prediabetes, and prevent disease progression.

The concept of “preventive treatment of disease” is a cornerstone of TCM, encapsulating its distinctive approach to health preservation. This philosophy emphasizes taking preventive measures before the onset of illness, intervening at the early stages of disease, and preventing its deterioration. Within TCM theory, an individual’s constitution is understood to form through a combination of innate and acquired factors, which collectively influence susceptibility to certain diseases (10). According to the Classification and Determination Standards of TCM Constitution issued by the China Association of Chinese Medicine on 9 April 2009 (11), the constitutions of the Chinese population are categorized into nine basic types: balanced constitution (BC), qi-deficiency constitution (QDC), yang-deficiency constitution (YDC), yin-deficiency constitution (YIDC), phlegm-dampness constitution (PDC), damp-heat constitution (DHC), blood-stasis constitution (BSC), qi-stagnation constitution (QSC), and inherited special constitution (ISC). Among these, the BC is considered the normal or ideal state, while the other eight are regarded as “unbalanced” and predispose individuals to related health conditions (12).

The core pathophysiological alteration in prediabetes resides in the dynamic imbalance between the progressive exacerbation of insulin resistance and the dysregulation of the compensatory insulin-secretion function of pancreatic islet β-cells (13, 14). Accumulating evidence indicates that IGR subjects with PDC, DHC, or QDC have high levels of insulin resistance and inflammatory response. IGR subjects with PDC or DHC are at a higher risk of developing diabetes, and such constitutions may therefore serve as potential predictive biomarkers for identifying individuals with impaired glucose regulation at high risk of developing overt diabetes, enabling targeted preventive interventions (15). The internal correlation between the constitution and pathophysiology offers a core theoretical foundation for intervening in prediabetes from the perspective of TCM constitution. In recent years, community health service centers across China have conducted extensive clinical studies on the relationship between prediabetes and TCM constitution. For example, based on the results of constitution identification using the TCM Constitution Scale (16), TCM practitioners provide personalized health prescriptions to guide patients in diet, daily routine, physical activity, and emotional regulation during the early stages of the diseases (17, 18). These interventions aim to achieve optimal health promotion and have been shown in several studies to effectively prevent or delay the progression to diabetes. Therefore, the purpose of this study is to synthesize existing evidence through a systematic review and meta-analysis, quantitatively assess the pooled prevalence of various TCM constitutions in the prediabetes population, identify common and high-risk constitution types, and provide evidence-based support for the early prevention and management of diabetes.

## 2 Materials and methods

### 2.1 Registry

The protocol was registered at PROSPERO (CRD42024607164) on 4 November 2024.

### 2.2 Retrieval strategy

Clinical studies on the correlation between TCM constitution and prediabetes were searched in PubMed, Embase, Web of Science, the Cochrane Library, China National Knowledge Infrastructure Database (CNKI), China Biomedical Literature Database (SinoMed), China Science and Technology Journal Database (VIP), and Wanfang Data from April 2009 to July 2025. The keywords searched are provided in **Supplementary Table S1** of the **Supplementary Materials**. Preferred Reporting Items for Systematic Reviews and Meta-Analyses (PRISMA) was used to construct the report of the current study (19) and the completed checklist is provided in the **Supplementary Materials** in **Supplementary Table S2**.

### 2.3 Inclusion/exclusion criteria

Inclusion criteria (1): Study subjects: a study of people diagnosed with prediabetes (2). Study design: all Chinese or English clinical studies on the relationship between TCM constitution and prediabetes (including cross-sectional studies and case–control studies) (3). Physique measurements are in accordance with the classification and evaluation standard of the TCM constitution issued by the Chinese Society of Traditional Chinese Medicine in 2009, and the article involves nine kinds of simple constitution (4). The physique data are complete.

Exclusion criteria (1): Lack of basic information reports or statistics on physical composition (2). Other systemic and serious diseases that may affect the types of medical physique (3). Limited to studies of specific types of physique, such as simple PDC research (4). Repeatedly published research data (5). Unable to obtain the full text (6). Reviews, reference papers, or literature on systematic reviews and meta-analyses.

### 2.4 Quality evaluation and data extraction on methodology

After removing duplicate records using NoteExpress software based on titles and abstracts, two researchers (Liqian Chen and Yun Wang) independently screened the literature, extracted data, and performed cross-verification. Any disagreements were resolved through adjudication by a third researcher (Wenying Wang). A standardized data extraction form was developed, which included information such as the literature title, study design, and geographical region. The methodological quality of the included case–control studies was assessed using the Newcastle–Ottawa Scale (NOS) (20). This tool evaluates studies across three domains: selection of study groups, comparability of groups, and ascertainment of exposure, with a total possible score of nine points. A score greater than six points indicates high quality (21). For cross-sectional studies, we used the assessment standard recommended by the Agency for Healthcare Research and Quality (AHRQ), which consists of 11 items (22). A score of 0–3 denotes poor quality; 4–7, fair quality; and 8–11, high quality. For consistency in this study, we defined a score greater than six points as indicative of high quality for both study types.

### 2.5 Data analysis

All statistical analyses were performed using Stata 17.0. We calculated the pooled prevalence and corresponding 95% confidence intervals (CIs) for each of the nine TCM constitution types within the prediabetes population. To investigate substantial heterogeneity, we conducted subgroup analyses based on region, age, and study quality. Based on the mean age of participants reported in each study and the WHO’s definition of older adults (≥60 years), all included studies were categorized into 2 subgroups: mean age ≥60 years and <60 years. This standard is widely used in global public health research for result comparison. Based on seven geographical divisions and study distribution in China, the regions were combined into four sub-regions: North China, East China, West China, and South China. This method balances sample size and reflects the influence of geographical environments on TCM constitutions. Publication bias was assessed using funnel plots and Egger’s test, while the stability of the results was evaluated through sensitivity analysis. Meta-regression was performed to identify potential sources of heterogeneity in the distribution of constitution types across studies. Furthermore, we performed a comparative meta-analysis of the prediabetes group versus the general population. The association was expressed as odds ratios (ORs) with 95% CIs. The choice of statistical model was based on the degree of heterogeneity: a fixed-effects model was applied when *I*^2^ was ≤50%, and a random-effects model was used when *I*^2^ was >50%.

## 3 Results

### 3.1 Research process

**Figure 1:**
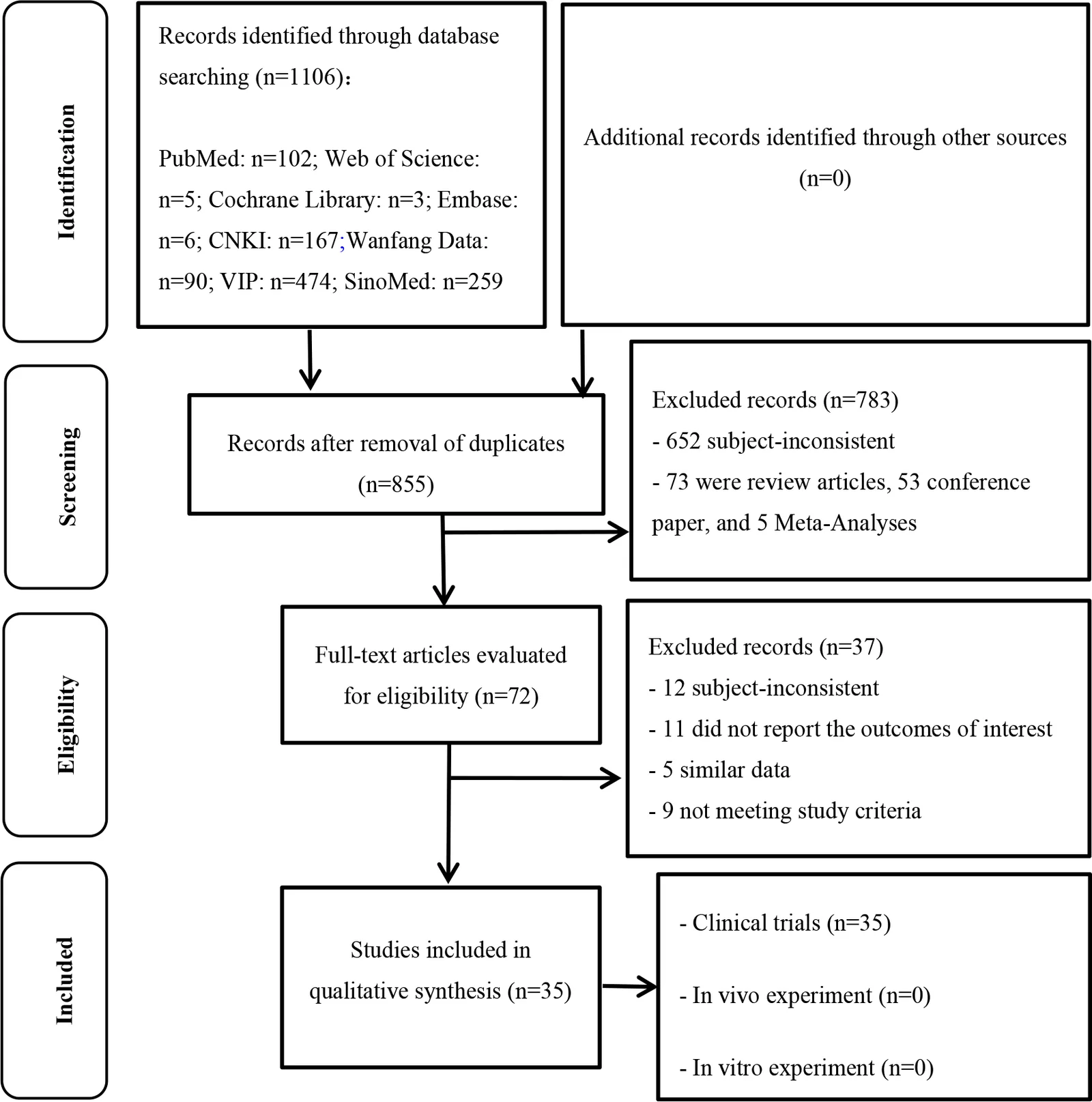
The flowchart of the screening and selection of studies.

After screening 1,106 studies found in literature retrieval, 72 papers may meet the inclusion criteria, and full-text examination was carried out. A total of 35 studies were included in the final review. The process and results of literature screening are shown in **Figure 1**.

### 3.2 Basic characteristics and quality evaluation of included studies

The systematic review included 35 studies, comprising 30 cross-sectional and 5 case–control studies. All were clinical studies conducted across 23 provinces, autonomous regions, and municipalities in China. The earliest study was published in 2011, with the number of publications increasing annually. In total, these studies included 8,469 individuals with prediabetes and 1,385 normoglycemic controls. Based on the NOS, three case–control studies were rated as high quality and two were rated as low quality. According to the AHRQ criteria, four cross-sectional studies were classified as high quality and 26 were classified as moderate quality. The basic characteristics of the included studies are summarized in **Table 1**.

**Table 1:** Characteristics of studies.

Study ID	Study time	Area	Study design	Sample size	Average age	Gender ratio (M/F)	Diagnostic criteria for prediabetes	Quality evaluation
Yang et al. (55)	2021.1–2021.12	Beijing	Css	85	48.58 ± 1 1.32	21/64	WHO	7
Li et al. (56)	2021.1–2021.8	Beijing	Css	450	47.02 ± 11.5	168/282	WHO	6
Wang (57)	2021.1–2023.11	Beijing	Css	248	NA	95/153	WHO	8
Liu (58)	2021.3–2021.11	Sichuan	Ccs	60 120	67.32 ± 9.1 64.9 ± 12.6	23/37 33/87	WHO	5
Chen et al. (59)	2015.1.1–2021.12.31	Guizhou	Css	420	73.242 ± 5.95	185/235	WHO	6
Wu et al. (60)	2021.11–2022.4	Dongguan	Css	200	29–74	113/87	WHO	5
Sheng et al. (61)	2018.10.20–10.27	Xinjiang	Css	87	NA	34/53	WHO	5
Yan et al. (62)	2019.2–8	Shanghai	Ccs	731 731	NA	258/473 230/501	ADA	6
Luan et al. (63)	2021.7–2022.1	Chengdu	Css	80	54	58/22	WHO	5
Chai et al. (64)	2019.12–2021.2	Xian	Css	181	48.72 ± 11.09	NA	WHO	5
Zheng et al. (65)	2019.3.1–2019.6.30	Fuzhou	Css	150	NA	73/77	ADA	4
Yang et al. (66)	2018.5–2020.5	Shantou	Css	500	47.32 ± 8.2	279/221	WHO	3
Zhou et al. (67)	NA	Shanghai	Css	180	65.03 ± 13.72	83/117	ADA	4
Zhang et al. (68)	2017.9–2018.10	Liaoning	Css	260	40.73 ± 12.17	116/144	WHO	7
Du et al. (69)	2011.1–2012.6	Shanghai	Css	294	52	156/138	WHO	8
Song et al. (70)	2015.1–6	Hebei	Css	120	55.49 ± 4.12	62/58	WHO	5
Wei et al. (71)	NA	Guangdong	Ccs	300 300	NA	NA	WHO	4
Sun et al. (72)	2016.8–2017.12	Xinjiang	Css	90	58.22 ± 11.98	41/49	ADA	5
Duan et al. (73)	2015.1–2017.1	Yunnan	Css	80	60.82 ± 2.1	62/18	WHO	5
Yu et al. (74)	2016.1–12	Tianjin	Css	486	52.1 ± 13.9	267/219	ADA	6
Hong et al. (15)	2011.1–2012.6	Shanghai	Css	306	52	165/141	ADA	9
Tan et al. (75)	2015.4–2016.11	Jiangsu	Css	60	54.48 ± 10.13	46/14	WHO	5
Zhang et al. (76)	2016.6–2017.8	Yunnan	Css	127	NA	NA	ADA	7
Sun et al. (77)	2015.3–2015.12	Guiyang	Css, ccs	153 156	58.84 ± 15.67 62.49 ± 13.79	66/87 72/84	WHO	8
Xu et al. (78)	NA	Zhuhai	Css	112	35–75	NA	WHO	4
Liu et al. (79)	2013.11–2014.11	Inner Mongolia	Ccs	104 104	49.07 ± 11.04 46.66 ± 11.62	60/44 49/55	ADA	6
Zhou et al. (80)	NA	Guizhou	Css	153	45.12 ± 2.9	106/47	WHO	4
Luo et al. (81)	2012.6–2012.12	Anhui	Css	295	47.30 ± 13.75	169/126	WHO	6
Gao et al. (82)	NA	Shaanxi	Css	134	57.22 ± 8.4	59/75	WHO	5
Tian et al. (83)	2011–2012	Shenzhen	Css	90	26–87	71/19	ADA	5
Sun et al. (84)	2011.11–2012.12	Tianjin	Css	478	25–75	NA	WHO	6
Zhang et al. (85)	2010.9–2011.8	Beijing	Css	147	55.49 ± 9.12	118/29	WHO	5
Zhang et al. (86)	2011.1–2012.1	Shenzhen	Css	517	43 ± 2.36	316/201	WHO	4
Li (87)	2010.7–2010.10	Shandong	Css	119	29–60	99/20	ADA	5
Han et al. (88)	2010.8–12	Beijing	Css	90	63.10 ± 9.12	74/16	WHO	5

Css, cross-sectional study; Ccs, case–control study; NA, not available; WHO, Word Health Organization; ADA, American Diabetes Association.

### 3.3 Meta-analysis of biased constitution distribution in TCM

A total of 35 studies, comprising 8,469 individuals, reported the distribution of TCM constitution types in the prediabetes population. The pooled proportion of each constitution type was calculated using a random-effects model due to significant heterogeneity (*I*^2^ > 50%). The analysis revealed that five constitution types had a prevalence exceeding 10%: PDC, BC, YIDC, QDC, and DHC. The results for these constitutions are presented in a forest plot, while the complete findings for all nine types are summarized in **Table 2**.

**Table 2:** Meta-analysis of the proportion of four other constitutions in prediabetes patients.

Constitution type	Sample size	Proportion(%)	95% CI	*p*	*I*^2^ (%)
Yang-deficiency	603	7	0.05–0.09	<0.01	90.43
Qi stagnation	381	5	0.04–0.07	<0.01	86.72
Blood stagnation	380	5	0.03–0.06	<0.01	83.67
Inherited special	134	1	0.01–0.02	<0.01	73.41

#### 3.3.1 Phlegm-damp constitution

**Figure 2:**
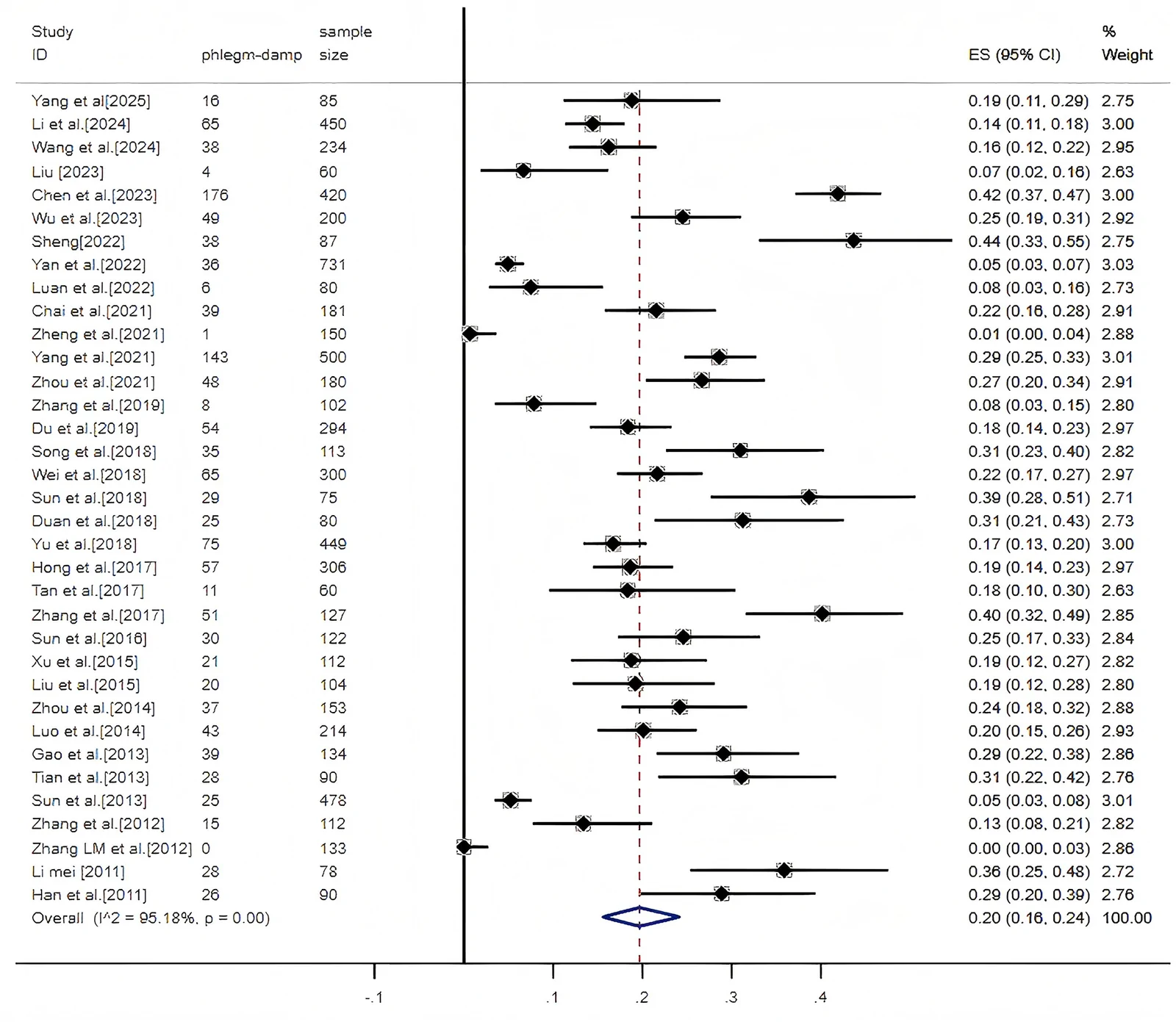
Meta-analysis of the proportion of PDC in patients with prediabetes.

A total of 35 studies, comprising 1,381 cases, reported the proportion of PDC in individuals with prediabetes. The meta-analysis demonstrated a pooled proportion of 20% (95% CI: 0.16–0.24, *p* < 0.01), as shown in **Figure 2**.

#### 3.3.2 Balance constitution

**Figure 3:**
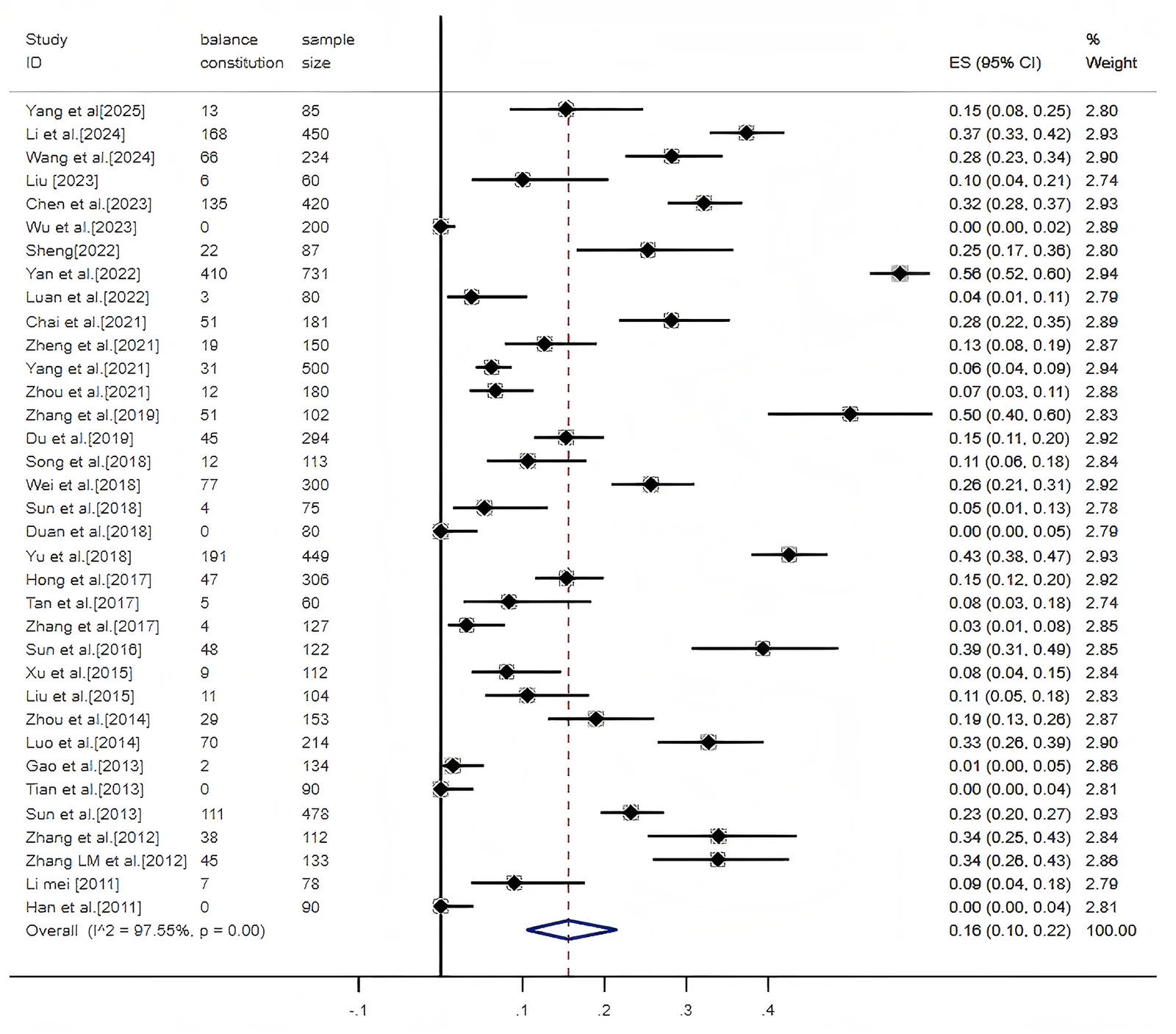
Meta-analysis of the proportion of BC in patients with prediabetes.

A total of 35 studies, comprising 1,742 cases, reported the proportion of BC in individuals with prediabetes. The meta-analysis demonstrated a pooled proportion of 16% (95% CI: 0.10–0.22, *p* < 0.01), as shown in **Figure 3**.

#### 3.3.3 Yin-deficiency constitution

**Figure 4:**
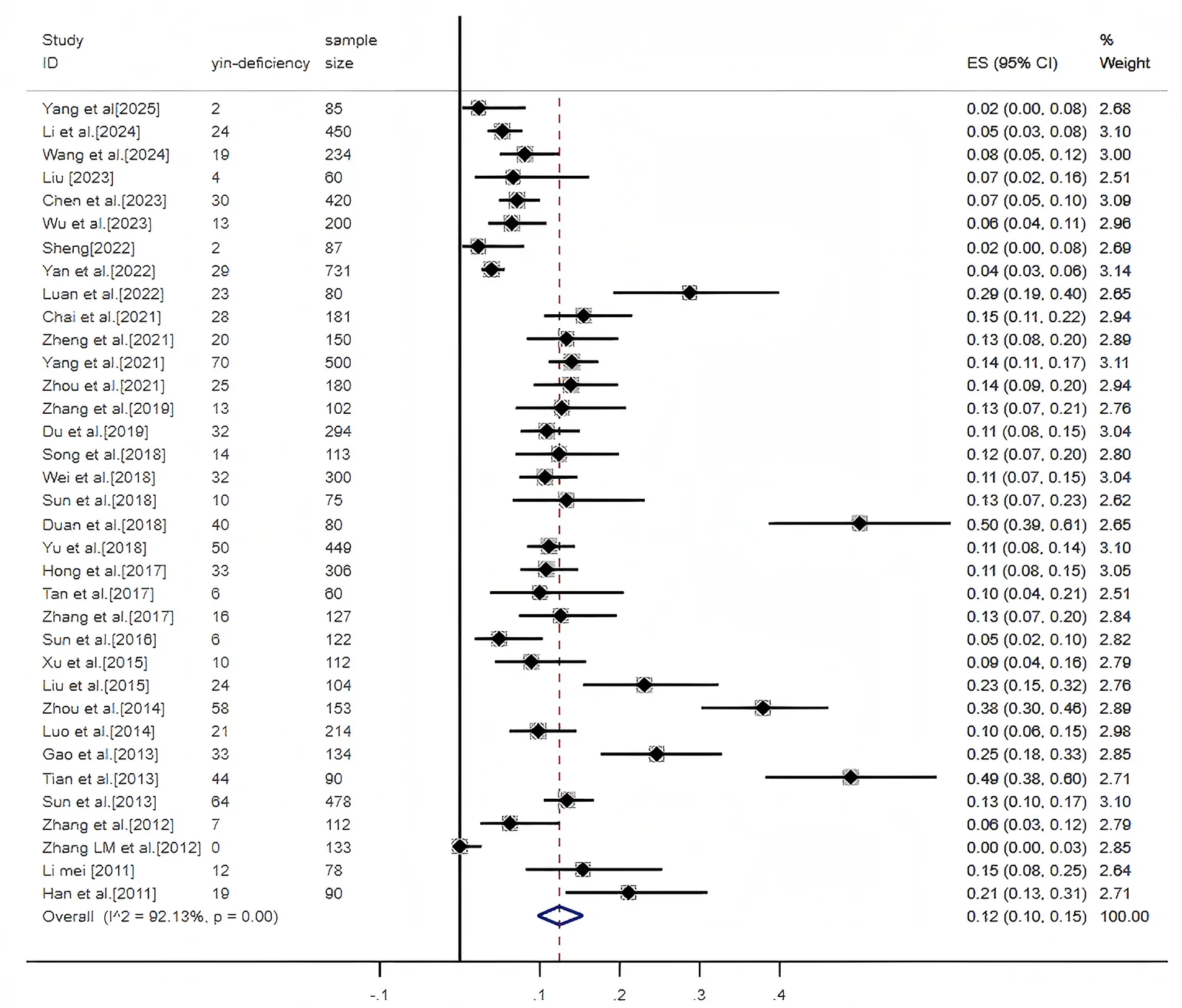
Meta-analysis of the proportion of YIDC in patients with prediabetes.

A total of 35 studies, comprising 833 cases, reported the proportion of YIDC in individuals with prediabetes. The meta-analysis demonstrated a pooled proportion of 12% (95% CI: 0.10–0.15, *p* < 0.01), as shown in **Figure 4**.

#### 3.3.4 Qi-deficiency constitution

**Figure 5:**
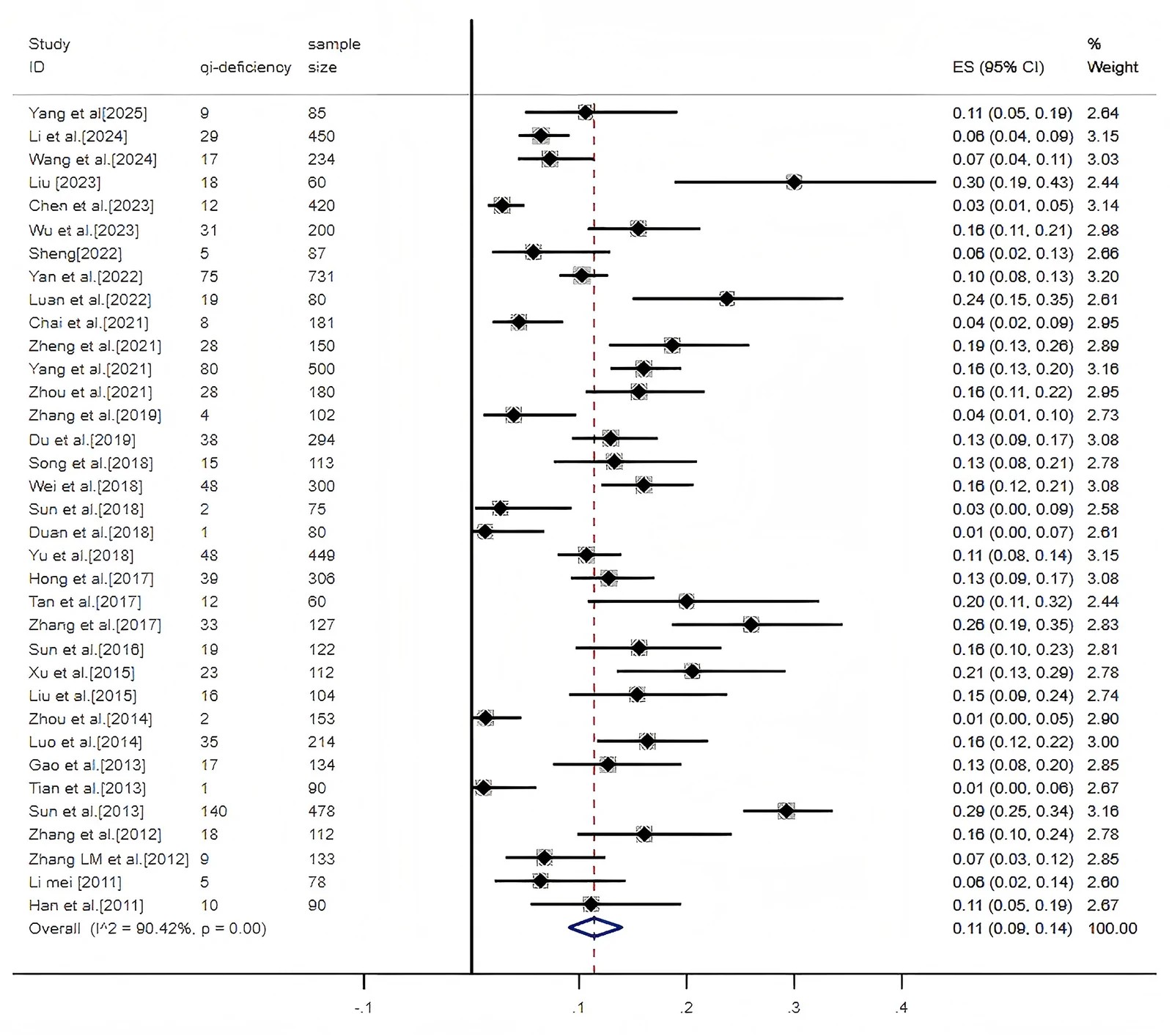
Meta-analysis of the proportion of QDC in patients with prediabetes.

A total of 35 studies, comprising 894 cases, reported the proportion of QDC in individuals with prediabetes. The meta-analysis demonstrated a pooled proportion of 11% (95% CI: 0.09–0.14, *p* < 0.01), as shown in **Figure 5**.

#### 3.3.5 Damp-heat constitution

**Figure 6:**
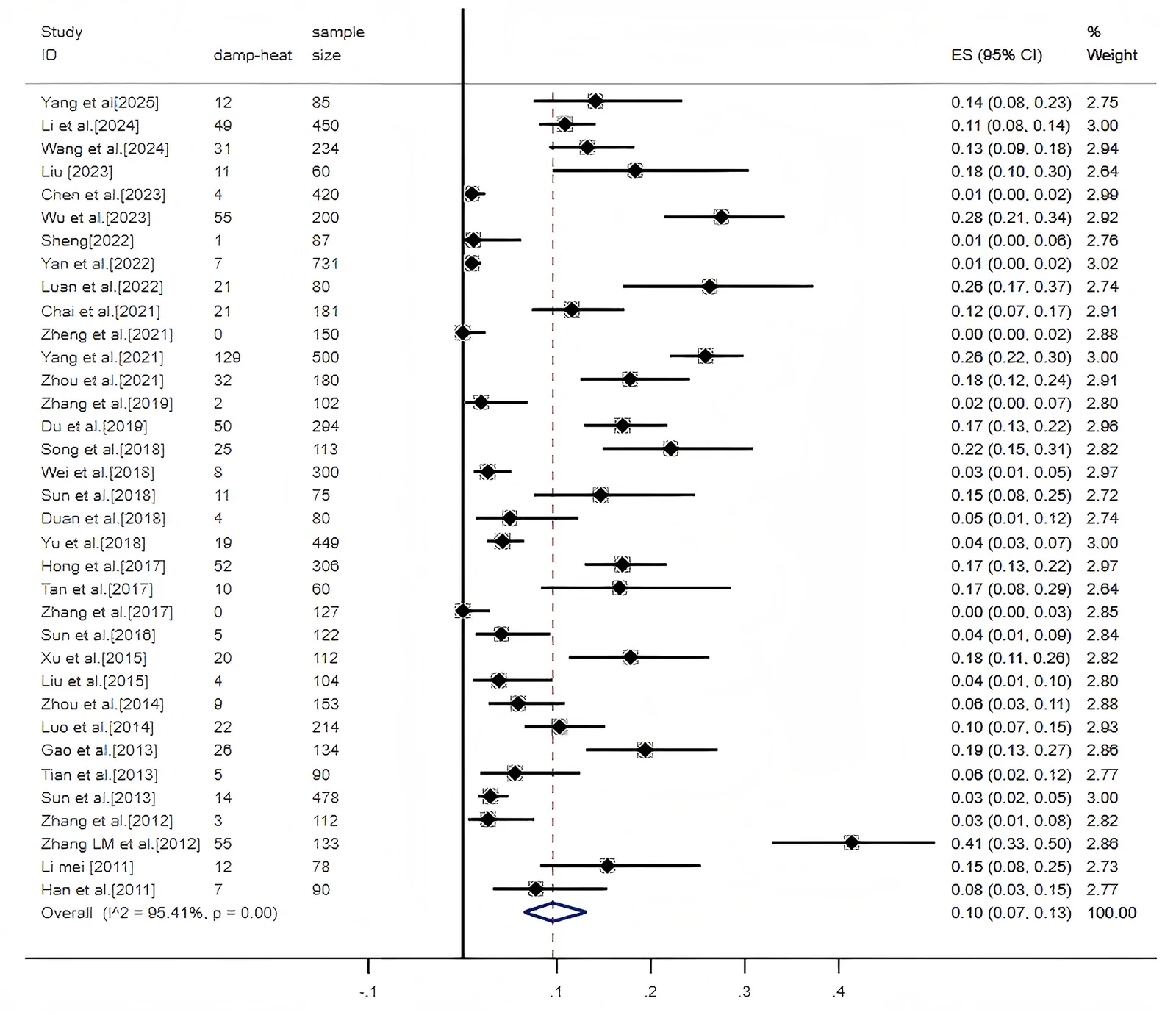
Meta-analysis of the proportion of DHC prediabetes patients.

A total of 35 studies, comprising 736 cases, reported the proportion of DHC in individuals with prediabetes. The meta-analysis demonstrated a pooled proportion of 10% (95% CI: 0.07–0.13, *p* < 0.01), as shown in **Figure 6**.

#### 3.3.6 Other TCM constitutions

The pooled proportion of four other types of TCM constitution in individuals with prediabetes was less than 10%. The order from high to low is YDC, BSC, QSC, and ISC (**Table 2**).

### 3.4 Meta-analysis of distribution of TCM constitution in patients with prediabetes and the general population

**Figure 7:**
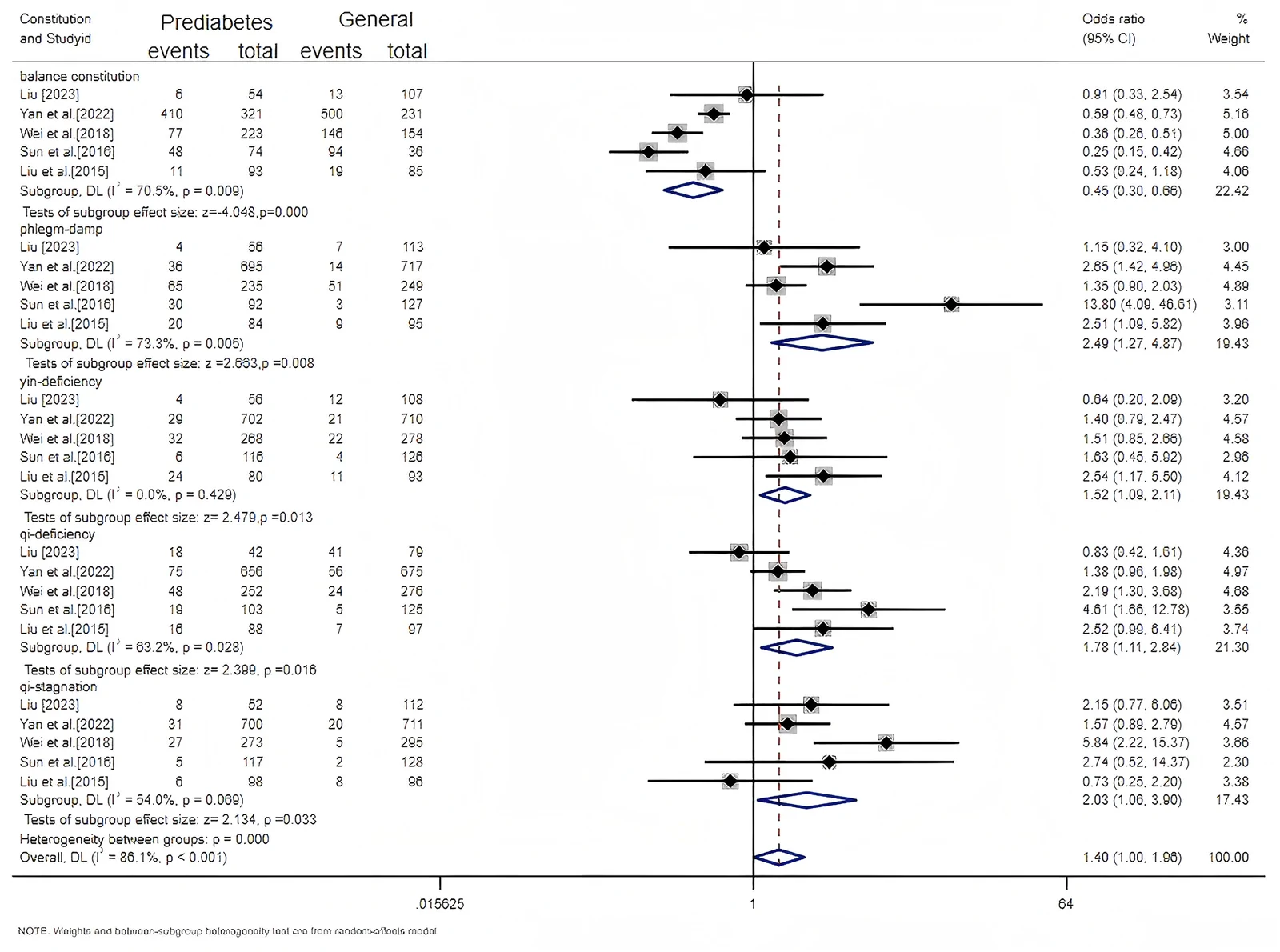
Comparison of five TCM constitutions’ distribution between patients with prediabetes and general population.

Five studies, involving 2,702 participants, provided comparative data. Meta-analysis revealed five TCM constitutions that were significantly associated with prediabetes: BC, YIDC, PDC, QDC, and QSC. The forest plot for this comparative analysis is presented in **Figure 7**.

Meta-analysis revealed significant differences in 5 TCM constitutions between patients with prediabetes and the general population. The ORs for prediabetes were as follows: PDC, 2.49 (95% CI: 1.27–4.87); QSC, 2.03 (95% CI: 1.06–3.90); QDC, 1.78 (95% CI: 1.11–2.84); YIDC, 1.52 (95% CI: 1.09–2.10); and BC, 0.45 (95% CI: 0.30–0.66). All these associations were statistically significant (*p* < 0.05). In contrast, no significant associations with prediabetes were observed for YDC, BSC, or ISC (all *p* > 0.05). Similarly, the proportion of DHC did not differ significantly between groups (OR: 1.06, 95% CI: 0.33–3.41) (**Table 3**).

**Table 3:** The nine constitutions between prediabetes patients and the general population.

Constitution type	Prediabetes	General	OR	95% CI	*I*^2^ (%)	*p*
BC	552	772	0.45	0.30–0.66	70.5	0.009
PDC	155	84	2.49	1.27–4.87	73.3	0.005
QSC	77	43	2.03	1.06–3.90	54.0	0.033
QDC	176	133	1.78	1.11–2.84	63.2	0.028
YIDC	95	70	1.52	1.09–2.10	0.0	0.012
YDC	124	169	0.67	0.47–1.06	52.7	0.076
ISC	46	26	1.57	0.68–3.62	42.9	0.136
BSC	57	41	1.34	0.60–2.98	59.5	0.478
DHC	35	47	1.06	0.33–3.41	80.7	0.914

### 3.5 Publication bias

**Figure 8:**
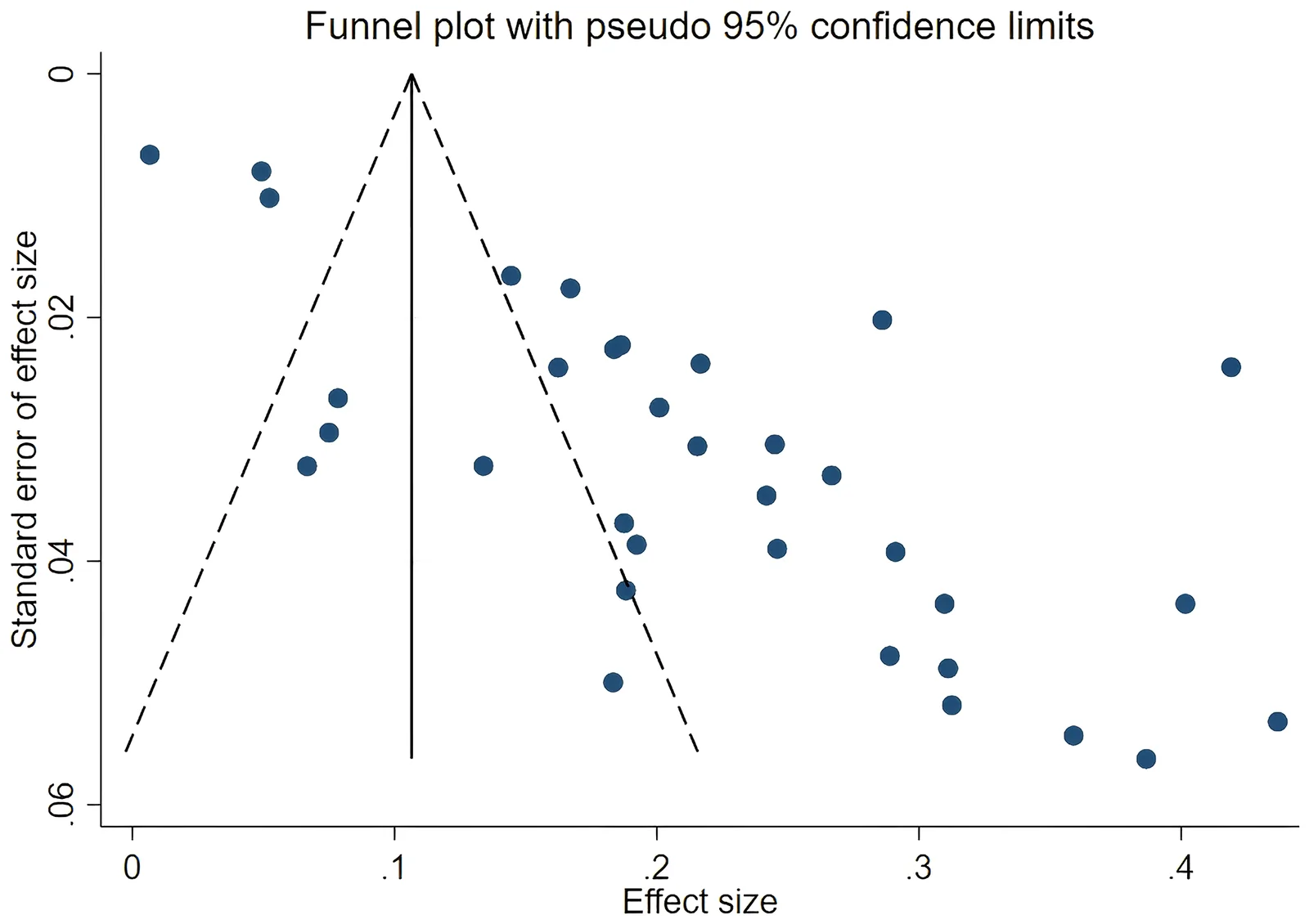
Funnel plot analysis of the distribution of PDC.

Publication bias was assessed using PDC, the most prevalent constitution, as an example. The funnel plot appeared asymmetrical (**Figure 8**), which is likely attributable to substantial clinical heterogeneity across studies. Such heterogeneity is common in TCM constitution research, which often involves diverse populations with variations in region, gender, and age, reflecting the inherent individual differences in these studies. This was supported by the results of Egger’s test (*t* = 8.10, *p* < 0.001) and Begg’s test (*z* = 2.49, *p* = 0.013), both indicating potential scattered state. A sensitivity analysis, performed by sequentially excluding each study, demonstrated that the pooled results for PDC remained robust, as no single study significantly altered the overall effect size (**Supplementary Figure S1**, **Supplementary Materials**).

### 3.6 Meta-regression analysis of the distribution of TCM constitution

**Figure 9:**
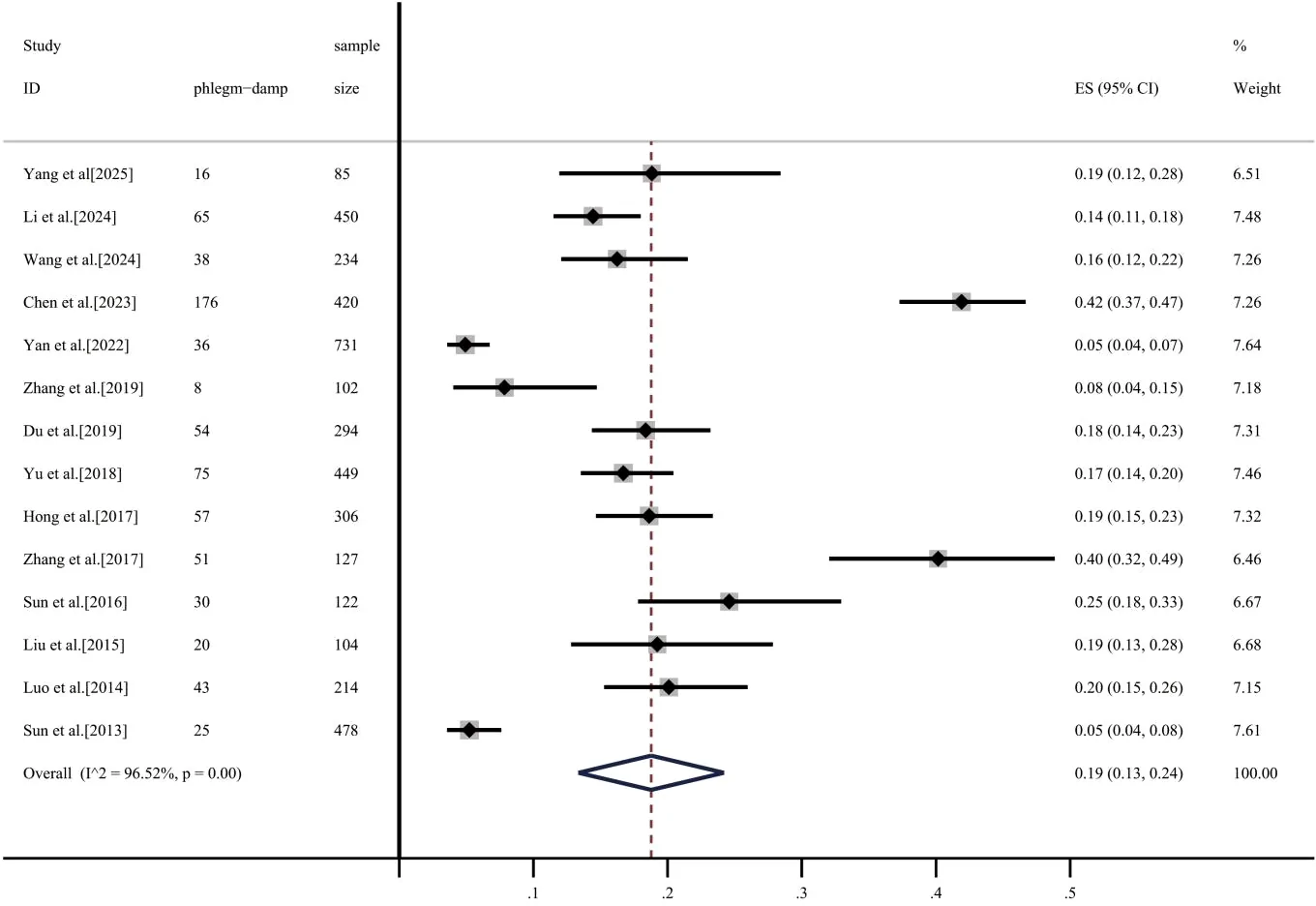
Sensitivity analysis of PDC after removing low-quality studies.

The sources of heterogeneity were investigated through sensitivity analysis, meta-regression, and subgroup analyses. Sensitivity analysis, performed by excluding low-quality studies (**Figure 9**), showed that the heterogeneity remained high (*I*^2^ = 96.52%), while the pooled prevalence of PDC decreased only slightly from 20% to 19%. Meta-regression was conducted using the following covariates: region, mean age, publication year, diagnostic criteria for prediabetes, and study quality (**Table 4**). The results indicated that these five covariates, when considered collectively, explained a portion of the substantial heterogeneity observed in the distribution of BC and DHC. Furthermore, the factors influencing the prevalence of different constitution types varied considerably. Specifically, mean age was positively associated with the prevalence of PDC, but not with other constitution types. Geographic region was identified as a significant factor influencing the prevalence of DHC. In addition, study quality emerged as a significant source of heterogeneity in the analyses of both BC and DHC.

**Table 4:** Summary of the results of meta-regression analyses.

Covariates	Mean age (β)	Region (β)	Study quality (β)	Diagnosis of prediabetes (β)	Year of publication (β)	*p* model	Adj *R*^2^ (%)	*I*^2^_res (%)
BC	−0.0045	−0.0248	−0.1504*	−0.0553	−0.0005	0.0380	30.42	99.97
PDC	0.0063*	−0.0027	0.0038	0.0268	−0.0088	0.2647	8.37	99.96
QSC	−0.0014	0.0016	0.0114	−0.0134	0.0053	0.8579	−15.72	99.89
QDC	0.0020	0.0102	0.0033	−0.0105	0.0224	0.8392	−15.03	99.93
YIDC	−0.0003	−0.0153	0.0967	0.0254	0.0004	0.6268	−6.86	99.96
DHC	−0.0038	0.0374*	0.0771*	−0.0145	−0.0198	0.0309	32.32	99.90
YDC	0.0023	−0.0130	−0.0284	0.0049	−0.0023	0.5686	−4.75	99.91
BSC	−0.0002	0.0070	0.0041	0.0278	0.0080	0.6758	−8.75	99.88
ISC	−0.0002	−0.0031	−0.0060	0.0078	−0.0018	0.4795	−1.31	99.30

*p < 0.05.

### 3.7 Subgroup analysis of the distribution of five common TCM constitutions of prediabetes

#### 3.7.1 Age

A random-effects model was applied owing to significant heterogeneity. Subgroup analysis revealed that among individuals with prediabetes, PDC was the most prevalent constitution type in both age groups. Moreover, the pooled prevalence of PDC was higher in the older age group (≥60 years) than in the younger group (<60 years). The meta-analysis results for the five most common constitution types are presented in **Table 5**.

**Table 5:** Meta-analysis of the proportion of five common constitutions of prediabetes in the age subgroup.

Constitution type	Age	Studies	Events	Proportion (%)	95% CI	*p*	*I*^2^ (%)
BC	<60	19	864	20	0.13–0.27	<0.01	96.38
	>60	5	279	6	0.00–0.23	<0.01	97.58
PDC	<60	18	722	18	0.14–0.23	<0.01	90.11
	>60	5	279	27	0.16–0.39	<0.01	91.29
DHC	<60	19	525	13	0.09–0.17	<0.01	93.02
	>60	5	58	8	0.02–0.19	<0.01	94.36
QDC	<60	20	419	11	0.08–0.13	<0.01	83.20
	>60	5	69	10	0.03–0.20	<0.01	93.47
YIDC	<60	18	454	12	0.08–0.16	<0.01	91.16
	>60	5	118	18	0.07–0.32	<0.01	94.90

#### 3.7.2 Region

All included studies were conducted in China. Studies were classified into these four regional subgroups, and the distributions of five common constitution types across these regions were compared (**Table 6**). The results indicated that PDC was the most prevalent constitution among patients with prediabetes in East China and South China, while DHC was the most common in South China.

**Table 6:** Meta-analysis of the proportion of five common constitutions of prediabetes in four regions.

Constitution	Area	Studies	Events	Proportion (%)	95% CI	*I*^2^ (%)	*p*
BC	North China	11	738	22	0.15–0.31	95.64	<0.01
	East China	6	538	17	0.04–0.37	98.74	<0.01
	West China	10	304	12	0.05–0.22	95.95	<0.01
	South China	4	162	8	0.01–0.21	97.53	<0.01
DHC	North China	12	200	8	0.05–0.12	87.74	<0.01
	East China	5	151	9	0.02–0.20	97.48	<0.01
	West China	10	113	8	0.03–0.14	92.74	<0.01
	South China	6	272	18	0.07–0.32	96.97	<0.01
PDC	North China	12	394	18	0.13–0.23	90.17	<0.01
	East China	6	207	13	0.05–0.23	96.25	<0.01
	West China	11	474	27	0.20–0.35	90.24	<0.01
	South China	5	306	18	0.09–0.30	95.92	0.05
QDC	North China	12	346	12	0.08–0.17	91.48	<0.01
	East China	6	220	14	0.11–0.17	60.13	0.03
	West China	11	136	9	0.04–0.16	92.43	<0.01
	South China	6	192	12	0.07–0.17	89.17	<0.01
YIDC	North China	12	269	11	0.08–0.14	80.65	<0.01
	East China	6	145	10	0.06–0.15	87.34	<0.01
	West China	11	250	16	0.09–0.25	94.19	<0.01
	South China	5	169	11	0.04–0.22	95.99	<0.01

#### 3.7.3 Study quality

To preliminarily explore the potential influence of study quality on the results, we classified studies into a high-quality group (score ≥6) and a low-quality group (score <6) based on their NOS scores, and calculated the pooled prevalence of each constitution type within these subgroups (**Table 7**). We observed a complex and varying relationship between study quality and constitution prevalence. Subgroup analysis indicated that the prevalence of BC was higher in high-quality studies, whereas for DHC, a higher prevalence was observed in low-quality studies.

**Table 7:** Subgroup analysis of five common constitutions regarding quality evaluation.

Constitution	Study quality	Proportion (%)	*I*^2^ (%)	95% CI	*p*
BC	High	27	97.19	0.19–0.36	0.00
	Low	9	94.85	0.05–0.14	0.00
PDC	High	18	96.44	0.12–0.25	0.00
	Low	21	93.30	0.15–0.27	0.00
QDC	High	12	93.60	0.08–0.16	0.00
	Low	11	86.80	0.08–0.14	0.00
DHC	High	6	94.85	0.03–0.10	0.00
	Low	13	94.10	0.08–0.18	0.00
YIDC	High	9	84.59	0.07–0.12	0.00
	Low	15	93.01	0.10–0.20	0.00

## 4 Discussion

Prediabetes is a metabolic condition characterized by elevated blood glucose levels and exhibits a high prevalence, particularly among older adults and individuals with obesity (23). Studies indicate that the aging of the Chinese population is a key factor driving the increasing prevalence of prediabetes (24). Additional risk factors include accelerated urbanization, sedentary lifestyles, and changes in living environments (25). However, the clinical symptoms of prediabetes are often subtle and easily overlooked, a condition that may be regarded as “no disease” in Western medicine. In contrast, clinical practice has demonstrated that TCM can significantly improve glycemic control and alleviate clinical symptoms in patients with prediabetes, effectively reducing the risk of progression to diabetes (26). Therefore, this meta-analysis holds significant implications for early screening and clinical intervention in prediabetes.

### 4.1 Analysis of the correlation between prediabetes and the TCM constitution

In our study, the most common TCM constitution types among individuals with prediabetes were PDC (20%), BC (16%), YIDC (12%), QDC (11%), and DHC (10%). These findings are consistent with a multi-center study (27), conducted in several cities including Shanghai, Nanjing, and Qingdao, which also identified PDC, DHC, and QDC as the most common constitution types among 1,590 patients with metabolic syndrome.

Case–control studies included in our analysis suggested that PDC, QSC, YIDC, and QDC may be risk factors for prediabetes, whereas BC appears to be a protective factor. Modern lifestyles characterized by high work pressure and irregular daily routines may contribute to the development of biased constitutions. PDC is a biased constitution primarily characterized by stickiness and turbidity, resulting from dysfunction in fluid metabolism, failure of the spleen to transport essence, and accumulation of phlegm and dampness (28). Individuals with PDC often present with oily skin, overweight, and a tendency toward drowsiness. Gene functional analyses on genes affecting the differences between PDC and BC indicated that people with PDC were susceptible to hyperlipemia and diabetes (29). Results of epidemiological surveys also show that PDC is associated with a higher risk of metabolic disorders such as hyperlipidemia, hyperuricemia, diabetes, and metabolic syndrome (29–32). Clinical studies further indicate that patients with impaired glucose regulation and PDC demonstrate elevated levels of inflammation, as evidenced by increased levels of interleukin-6 (IL-6) and tumor necrosis factor-α (TNF-α). Moreover, these patients exhibit reduced gut microbiota diversity, abnormal short-chain fatty acid metabolism, and heightened insulin resistance (15). Notably, PDC is closely linked to unhealthy lifestyle behaviors—such as a diet rich in greasy and sweet foods and insufficient physical exercise—which are established risk factors for metabolic syndrome and diabetes.

In TCM theory, qi is considered the fundamental substance constituting the human body and maintaining life activities (33). QDC is characterized by fatigue, weak voice, and a faint pulse. Its pathophysiology has been associated with intestinal microbiota dysbiosis and subsequent impairments in metabolic and immune functions, aggravating sugar metabolic disorder (34, 35). QSC, resulting from impaired qi flow, often manifests as emotional irritability or anxiety. Chronic stress may contribute to insulin resistance and type 2 diabetes via the dysregulation of the hypothalamic–pituitary–adrenal axis and elevated cortisol levels. These factors inhibit insulin secretion and promote hepatic glucose production, consequently increasing the risk of prediabetes (36, 37). Both QDC and QSC have been linked to diabetes, depression, and obesity (38–40). YIDC typically presents with symptoms of internal heat (38), dry mouth, and a relatively thin physique. Clinical studies have correlated this constitution with elevated fasting blood glucose and increased inflammatory markers. These markers induce inflammatory factors, stimulate the formation of oxygen free radicals, and promote the production of peroxidation metabolites (41, 42). These factors jointly contribute to pancreatic β-cell and insulin target tissue cell damage and impaired insulin signaling in target tissues, playing a significant role in the development of islet dysfunction and insulin resistance (43). In contrast, individuals with BC exhibit a more stable gut microbiota environment, with lower levels of immune-inflammatory factors and higher cardiopulmonary reserve function compared to those with PDC. They maintain a state of dynamic balance, characterized by abundant energy, overall health, emotional stability, and good sleep quality (44).

In the regional subgroup analysis, the prevalence of PDC and DHC was highest in South China, followed by West China. We speculate that these patterns are closely related to climatic and dietary factors. The hot and humid climate in South China is conducive to the development of dampness, phlegm, and heat. In West China, the local diet is characterized by spicy, greasy, and sweet foods, which may impair spleen and stomach function and lead to chronic deficiency of body fluids, resulting in a higher proportion of individuals with YIDC and PDC. Furthermore, in economically developed and relatively affluent regions, increased consumption of high-calorie fast food may contribute to obesity and the accumulation of phlegm-dampness (45, 46). Age-based subgroup analysis indicated a high proportion of PDC in both age groups. This may be attributed to dietary habits and lifestyle factors that disrupt metabolic homeostasis, predisposing individuals to biased constitutions. Additionally, with advancing age, physiological functions gradually decline, leading to various pathological changes such as insufficiency of body fluids and a tendency toward dampness accumulation and phlegm formation.

Meta-regression analysis revealed distinct associations of age, study quality, and geographical region with different constitution types. We observed a positive correlation between PDC and age, which aligns with physiological changes described in modern medicine—namely, the slowing of metabolism and decline in fluid regulation with aging. DHC was positively correlated with region, supporting the TCM principle of “correspondence between humans and nature”, and underscoring the influence of environmental factors such as climate and diet on the formation and distribution of DHC. Interestingly, while meta-regression indicated a negative correlation between study quality and BC, the subgroup analysis showed a higher prevalence of BC in high-quality studies. This discrepancy may be attributable to diagnostic inaccuracy and inadequate control of confounding factors in lower-quality studies, which could lead to misclassification and bias in constitution determination. Similarly, although DHC was positively associated with study quality in the meta-regression, subgroup results suggested the opposite. This inconsistency may reflect biases in studies with high reporting rates, such as broad diagnostic criteria or regional clustering. Future studies should incorporate detailed dietary patterns, finer geographical distinctions, and standardized quality assessments. Multi-center, large-scale collaborative research is warranted to further validate these findings.

Current clinical management of prediabetes primarily involves lifestyle interventions and health management, supplemented by both Western pharmacological approaches and TCM therapies. Early intervention based on TCM constitution theory has shown promise in delaying or preventing the progression from prediabetes to diabetes. For example, one clinical study implemented constitution-based differentiation and targeted TCM therapies in individuals with prediabetes, demonstrating improved blood glucose control and a reduction in diabetes incidence (47). Another study found that combining TCM with metformin resulted in greater reductions in body weight, fasting blood glucose, and total cholesterol compared to metformin alone, with no significant adverse effects reported (48). Various Chinese herbal medicines and formulations have also been investigated for glucose regulation. Astragalus (Huangqi), a traditional herb used to strengthen the spleen and replenish qi, contains active components such as astragalus polysaccharides, saponins, and flavonoids (49). Experimental studies have shown that Astragalus extracts exert anti-inflammatory and pancreatic repair effects, leading to improved pancreatic function and significant reductions in fasting blood glucose and food intake in diabetic mouse models (50). Clinical studies have further demonstrated that formulations such as Tangzhiping Decoction and Jinlida can alleviate insulin resistance and improve glucose and lipid metabolism disorders, thereby reducing the risk of type 2 diabetes (26, 51, 52).

TCM has been validated through millennia of clinical practice and is increasingly supported by modern research. Guided by constitutional theory and supplemented with modern diagnostic techniques, TCM approaches—including dietary modification, acupuncture, and herbal medicine—can help correct biased constitutions. These strategies align with the three-level preventive model in TCM: preventing disease before it arises, treating it at early stages, and mitigating its progression (53, 54).

### 4.2 Limitations of the study and implications for the future

Most of the included studies were cross-sectional in design, which limits the ability to establish causal relationships between TCM constitution types and prediabetes. Furthermore, the majority of articles were of moderate to low quality, and important confounding variables—such as dietary habits, comorbidities, and external environmental factors—were not consistently controlled or included in the meta-analysis. These limitations may have contributed to the significant heterogeneity observed and potential publication bias. To strengthen the evidence base, future research should prioritize high-quality, prospective cohort designs, multi-center collaborations, and large-sample studies. The TCM Constitution Scale could serve as a valuable tool for identifying individuals with high-risk constitutions—such as PDC, YIDC, QDC, and QSC constitutions—enabling early intervention to correct constitutional bias and reduce the risk of progression to diabetes. In clinical practice, treatment strategies should consider not only pathological factors but also physiological and constitutional characteristics across different age groups.

## 5 Conclusion

This systematic review of 35 studies indicates that the most common TCM constitution types in individuals with prediabetes are PDC, BC, YIDC, QDC, and DHC. Among these, PDC, QDC, YIDC, and QSC may represent risk factors for prediabetes, whereas BC appears to be a protective factor. For future prevention and clinical management, early identification of high-risk populations—such as older adults with PDC in South China and West China—is recommended. Targeted interventions, including acupuncture and TCM-based therapies, may help correct constitutional imbalances and facilitate a return to a balanced state. Moreover, the TCM Constitution Scale offers a non-invasive and cost-effective method for screening high-risk groups in clinical settings, supporting early intervention and personalized diabetes prevention strategies.

## Data Availability

The datasets presented in this study can be found in online repositories. The names of the repository/repositories and accession number(s) can be found in the article/**Supplementary Material**.
